# inGAP-family: Accurate Detection of Meiotic Recombination Loci and Causal Mutations by Filtering Out Artificial Variants due to Genome Complexities

**DOI:** 10.1016/j.gpb.2019.11.014

**Published:** 2021-03-10

**Authors:** Qichao Lian, Yamao Chen, Fang Chang, Ying Fu, Ji Qi

**Affiliations:** State Key Laboratory of Genetic Engineering, Institute of Plant Biology, School of Life Sciences, Fudan University, Shanghai 200433, China

**Keywords:** inGAP-family, Genomic variation, Structural variation, Meiotic analysis, Genetic mapping, Causal mutation

## Abstract

Accurately identifying DNA polymorphisms can bridge the gap between phenotypes and genotypes and is essential for molecular marker assisted genetic studies. Genome complexities, including large-scale **structural variations**, bring great challenges to bioinformatic analysis for obtaining high-confidence genomic variants, as sequence differences between non-allelic loci of two or more genomes can be misinterpreted as polymorphisms. It is important to correctly filter out artificial variants to avoid false genotyping or estimation of allele frequencies. Here, we present an efficient and effective framework, **inGAP-family**, to discover, filter, and visualize DNA polymorphisms and structural variants (SVs) from alignment of short reads. Applying this method to polymorphism detection on real datasets shows that elimination of artificial variants greatly facilitates the precise identification of meiotic recombination points as well as **causal mutations** in mutant genomes or quantitative trait loci. In addition, inGAP-family provides a user-friendly graphical interface for detecting polymorphisms and SVs, further evaluating predicted variants and identifying mutations related to genotypes. It is accessible at https://sourceforge.net/projects/ingap-family/.

## Introduction

DNA polymorphisms are genetic variations among individuals, including single nucleotide polymorphisms (SNPs), small insertions and deletions (indels), and large-scale structural variants (SVs) [1], [2], [3], [4]. They may affect some key cellular functions, resulting in severe phenotypic consequences in the growth and development of humans, animals [5], [6], and plants [7]. These polymorphisms also provide abundant molecular markers for genetic researches covering various aspects including but not limited to: revealing meiotic recombination points using tetrad analysis upon parental and meiotic products; identifying causal mutations from high-throughput sequencing of populations or pooled meiotic progenies (*e.g.*, F_2_ progenies) in genotyping-based studies [8], [9], [10], [11].

Many efforts have been made to develop effective bioinformatics methods for identifying polymorphisms from high-throughput sequencing data, *e.g.*, SAMtools [12] and GATK [13]. Then specific genomic regions or genes harboring causal mutations responsible for particular phenotypes can be predicated by using tools such as SHOREmap [14] and MutMap [15] on mutants of model species [9], [14], [15], [16], including natural mutants or artificial mutants induced by ethane methyl sulfonate (EMS), T-DNA insertion, or others. However, accurate and efficient identification of polymorphisms can still be challenging due to the complex nature of genomes and the limitation of next-generation sequencing (NGS) technologies. For example, human, animal, and plant genomes usually contain many repetitive sequences that differ in copy number among populations. Incorrectly mapped reads of highly similar non-allelic sequences can yield false polymorphism calls and genotypes [17], [18], [19]. In studies sensitive to false positive polymorphism callings, *e.g.*, detection of meiotic recombination loci by comparing outcrossing species, the number of gene conversions (GCs) could be much overestimated if artificial polymorphisms are included [18], [20]. Furthermore, due to the limitation of sequencing technologies, the short length of sequencing reads and their uneven coverage on reference genomes particularly in high GC content and repetitive regions [4], [18], [21], [22], [23] would unavoidably affect the precision in identifying genetic variants [22]. Therefore, to meet the challenge brought by genome complexities and sequencing technologies, a more efficient analytic strategy is needed for accurate discovery of genetic variations, polymorphism-dependent genotyping, and identification of causal mutations.

In this study, we present a framework, inGAP-family, for obtaining high-confidence genomic variant callings from resequencing data of single individuals, family-based samples, or pooled populations, by filtering out artificial variants caused by the false mapping of non-allelic short reads associated with large-scale SVs and tandemly repeated regions. Based on highly confident polymorphism markers discovered, inGAP-family could be further applied to detect meiotic recombination events from progeny genomes or to identify causal mutations in genotypic studies. Compared with SAMtools [12], GATK [13], and SHOREmap [14], inGAP-family displays better performance on real sequencing datasets for studies of meiotic recombination and genetic mapping, facilitated with a user-friendly graphical interface.

## Method

The inGAP-family is designed for accurate detection of polymorphisms and identification of meiotic recombination events and causal mutations from resequencing data of specific individuals, family-based samples, or pooled populations. As shown in Figure S1, inGAP-family employs a three-step strategy to obtain accurate polymorphisms through stringent filtering procedures: 1) identify multiple types of polymorphisms, including SNPs, small indels, and large-scale SVs, from mapping information of paired-end reads as described in previous studies [24], [25]; 2) filter out artificial SNPs and indels by considering various possibilities, including mapping of non-allelic sequences and incomplete coverage of reads on tandemly repeated regions or other SVs (which may differ among genomes of different individuals or ecotypes) [18]; and 3) automatically apply the high-confidence SNPs and indels on multiple analyses related with genotyping, *e.g.*, detection of crossovers (COs) and GCs and identification of causal mutations or quantitative trait loci (QTLs). The predicted polymorphisms can be examined through a graphical interface of inGAP-family.

### Predicting variants including SNPs, small indels, and large-scale SVs

Most artificial polymorphisms are caused by sequencing errors, incomplete coverage of tandem repeats, or false mapping of non-allelic reads. These artificial polymorphisms, if are recognized as genomic markers, will bring a destructive effect on those studies, which are sensitive to rare genomic loci, *e.g.*, detection of meiotic recombination points [18], [20]. Therefore, it is of crucial importance to consider the impacts brought by genome complexities on SNP and small indel calling [18]. To obtain high-confidence SNPs and small indels, chromosomal context must be consulted, especially when involving copy number variants (CNVs) or other types of large-scale SVs. inGAP-family adopts a Bayesian-based strategy [24] to detect candidate SNPs and small indels from mapped short NGS reads. Non-uniquely mapped reads with mapping quality score lower than 20 (provided by BWA aligner) are ignored for the procedure of polymorphism calling. inGAP-family also filters out reads with mapping ends staying in tandemly repeated regions, which are recognized by Tandem Repeats Finder [26] with a minimal alignment score of 10 and maximum period size of 20. Furthermore, various types of SVs, including large indels, inversions, translocations, and CNVs, are detected by inGAP-family based on the distance and the direction of paired ends and the information of coverage depth and split reads [25].

### Refining variants by examining potential mistakes

To eliminate the unreliable variants, predicted polymorphisms need to be thoroughly evaluated by the following procedures. First, SNP and small indel candidates are filtered out if located within or adjacent to large-scale SVs, which lead to false mapping of non-allelic paralogous sequences [18]. Small indels are further examined for gain/loss of tandem units when overlapped with tandemly repeated regions. Heterozygous indels contributed by reads without fully covering tandemly repeated regions are ignored. Second, considering that CNVs with complex patterns of paired-end read mapping could be missed in the procedure of SV detection, distribution of read coverage for each SNP candidate is further examined for potential CNVs. A polymorphism is ignored when it meets two requirements (default parameters): its read coverage exhibits four-fold or higher coverage than the average sequencing depth; its read coverage falls in range of top 1% or bottom 1% percentile of the distribution of all polymorphism candidates. For heterozygous variant callings in diploid genomes, an extra examination is performed: a qualified polymorphism must be supported by expected allelic ratio (99% percentile of the distribution of total polymorphisms), which is defined as the relative percentage of reads with the primary allele to the sum of reads with either primary or alternative alleles. When two or more samples are involved in a single analysis, polymorphisms are called and filtered for each sample independently, and are then merged into a single file with VCF format, in which genotype of each sample on each polymorphic locus is re-evaluated.

### Automatic discovery of meiotic recombination events by utilization of the high-confidence polymorphisms

inGAP-family employs a graphical interface to detect meiotic recombination events (COs and GCs) from high-throughput resequencing data of meiotic progeny genomes. Input files include: a reference genome, which does not have to be one of the parental genomes; a set of high-quality SNPs and indels between two parental genomes (as described above) for genotyping procedures; mapping files of high-throughput sequences of progenies against the reference genome. Each uniquely mapped read of a progeny genome is genotyped if overlapped with one or more polymorphism loci. Then allelic ratio is calculated for each SNP as follows:

$${\mathrm{Ratio}}_{s}=\frac{M_{\mathrm{supp}}}{P_{\mathrm{supp}}+M_{\mathrm{supp}}}$$

where $P_{\mathrm{supp}}$ is the number of paternal-allelic reads and $M_{\mathrm{supp}}$ is the number of maternal-allelic ones. As the allelic ratio on a single nucleotide locus randomly fluctuates along with sequencing depth, inGAP-family employs sliding windows (size of 20 kb with step of 10 kb) to smooth the estimation of ratio for genotyping and recognition of CO boundaries. inGAP-family adopts an empirical formula for choosing window size, considering SNP density and sequencing depth:

$$\mathrm{Window}\;size=\frac{2\times 10^{3}}{\mathrm{Sequencing}\;depth}\times \frac{\mathrm{Genome}\;size}{\mathrm{SNP}\;number}$$

The window size is approximately 20 kb for resequencing data with 20× depth on a genome of 100 Mb when having an SNP density of one substitution per 200 bp between ecotypes. Allelic ratio of a window is determined by:

$${\mathrm{Ratio}}_{w}=\frac{\sum_{i}P_{i}}{\sum_{i}\left(\left(P_{i}+M_{i}\right)\right)}$$

in which $P_{i}$ and $M_{i}$ denote the paternal-allelic and maternal-allelic read numbers of the *i*-th SNP in the window, respectively. The genotype calling of a window is then defined as:

$$G_{w}=\left\{\begin{array}{c} \mathrm{Parental}\;homozygous,\;{\mathrm{when}\;Ratio}_{w}\geq 0.8\\ heterozygous,\;when\;{0.2<Ratio}_{w}<0.8\\ Matenal\;homozygous,\;when\;{\mathrm{Ratio}}_{w}\leq 0.2 \end{array}\right)$$

Adjacent windows with the same genotype (paternal homozygous, maternal homozygous, or heterozygous) are grouped into larger blocks for each chromosome. Positions of COs are identified when genotypes switch between neighboring blocks, and are refined by examining the genotypes of flanking SNPs. Furthermore, the interface of inGAP-family allows users to manually modify CO boundaries or import a list including positions and genotypes of CO blocks. A SNP with allelic ratios inconsistent with the background genome is marked as a potential GC locus, and adjacent converted SNPs are grouped into one GC event.

### Recognition of causal mutations in the studies of QTLs and genetic mapping

inGAP-family identifies causative mutations from bulked DNA of multiple progenies with same phenotype at a single-base resolution. In the studies of genetic mapping, mutations are induced on one ecotype by EMS, T-DNA insertion, or others. F_2_ progenies hybridized from the mutant and another ecotype (a wild type) are selected for pooled sequencing when they exhibit the given phenotypes. As mentioned in the section of recombination event detection, high-quality polymorphisms are used as genetic markers between the two parental lines. Each read of F_2_ progenies is genotyped from mapping results against the reference genome sequence if overlapped with SNPs or indels. Sliding windows with size of 200 kb and step of 100 kb are applied for estimating the allelic ratio ${\mathrm{Ratio}}_{w}$, as described above. Here, choosing proper window size needs to take into account of genome size, crossover frequency per meiosis, and number of F_2_ progenies employed. Allelic ratios of windows are further smoothed, by employing longer sliding windows (size of 2 Mb and step of 500 kb), to calculate first-order and second-order derivatives for obtaining extreme values (peaks or valleys), which represent candidate regions carrying causal mutations. The predicted ranges of candidate regions associated with causal mutations can be refined through the graphical interface of inGAP-family when needed. Furthermore, inGAP-family identifies novel SNPs on peak/valley regions for EMS-induced mutants, then performs: 1) filtering out heterozygous SNPs whose allelic ratio smaller than 0.75; 2) excluding SNPs whose alternate allele is consistent with parental genomes; and 3) extracting SNPs that exhibit transitions from ‘G’ to ‘A’ or from ‘C’ to ‘T’. In addition, inGAP-family can also identify causal insertions by prediction and scanning of novel SVs on candidate regions for mutants induced by T-DNA insertion. Detected mutations are then annotated according to their annotation models (exon, intron, intergenic region, 5′-UTR, 3′-UTR, *etc.*), and are further classified as synonymous or non-synonymous when located in coding regions as potential for change- or loss-of-functions.

### Dataset 1

To evaluate the performance of inGAP-family, we downloaded the whole genome sequences of two ecotypes of *Arabidopsis thaliana*, Columbia (Col, SRX202246, 9.6 Gb, ∼ 80× coverage) and Landsberg *erecta* (L*er*, SRX202247, 8.4 Gb, ∼ 70× coverage) [20]. The two datasets consist of 2 × 100 bp paired-end reads with an average insert size of 500 bp sequenced on an Illumina HiSeq 2000 platform. A list of SNPs and small indels between Col and L*er* is downloaded from the 1001 Genomes project website (http://1001genomes.org/projects/MPISchneeberger2011/index.html) for evaluation of variant calling by different methods. The genome of a meiotic progeny of the two ecotypes (an F_2_ plant, SRX202243, 11.3 Gb, ∼ 95× coverage) was introduced to test the performance of inGAP-family on uncovering meiotic recombination events. Short reads of Col, L*er*, and the meiotic progeny genome were aligned to the TAIR10 assembly of *A. thaliana* (Col) reference genome [27] by BWA (version 0.7.12) [28].

### Dataset 2

We also tested the effectiveness of inGAP-family on detection of causal mutations based on genetic mapping. We obtained an EMS-induced *A. thaliana* mutant in L*er* ecotype, carrying a recessive mutation on the *ABA DEFICIENT 3* (*ABA3*, AT1G16540) gene involved in abscisic acid biosynthesis and salt tolerance. The mutant was backcrossed with wild-type Col, and 100 F_2_ progenies with salt-tolerance phenotype were selected for pooled DNA sequencing. The bulked DNA was sequenced on an Illumina HiSeq 2000 platform, providing 44 million 101-bp paired-end reads (3 Gb, ∼ 25× coverage). High-confidence polymorphisms between Col and L*er* were obtained as described above. Short reads were aligned to the TAIR10 reference genome using BWA [28], and the mapping results were adopted as input of inGAP-family for detecting causal mutations.

## Results

### Genome complexity leads to artificial SNP callings from non-allelic or repetitive regions

Eukaryotic genomes usually contain large amount of repetitive sequences including transposable elements (TEs), gene duplicates, and tandemly repetitive oligonucleotides that may differ in copy number among species. The genomic SVs must be carefully considered for calling of polymorphisms and phenotype detection. The model plant *A. thaliana*, which is widely used for genetic and genomic analyses on gene functions [1], has a great number of SVs including deletions, insertions, and CNVs between two ecotypes, Col and L*er*, revealed by many resequencing data [20], [29], [30].

**Figure 1:**
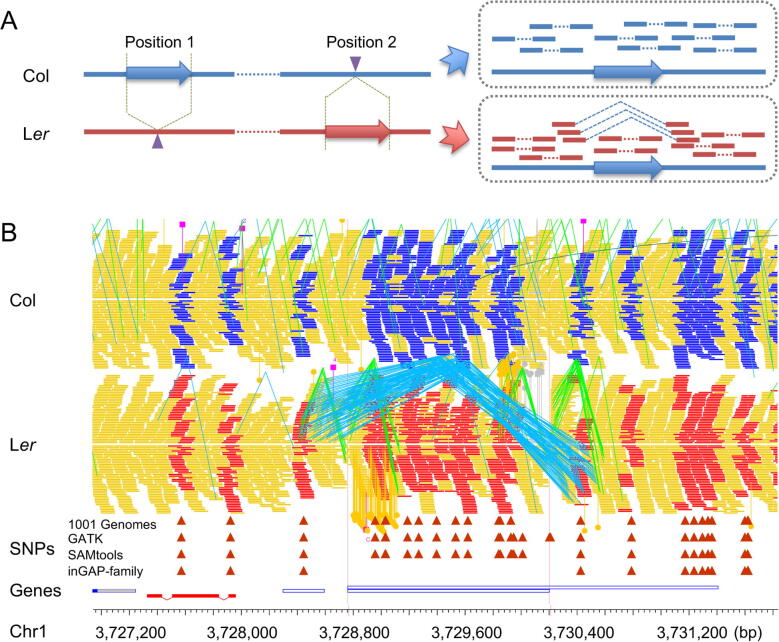
**Transposition between two genomes affects mapping of non-allelic sequences and genotype evaluation** **A.** Illustration of detected transpositions between Col and L*er*. On the left, large arrows denote paralogous sequences between the two genomes, while Col sequences are colored in blue and L*er* in red. On the right, read depth, mapping distance, and orientation of paired-end reads are shown around an SV. L*er* read bars linked by blue lines (exhibiting longer distance than average) indicate a deletion, and the reads within the deletion are from non-allelic positions, while the others are mapped normally. **B.** An example of transposition between Col and L*er* (3,728,969–3,730,413 bp on Chr1 of Col) analyzed from resequencing data. Col reads (blue bars) and most of L*er* reads (red bars) are mapped normally. The paired-end reads of L*er* with blue lines denote a deletion and the reads on the region (a TE) are falsely mapped paralogous sequences. Col or L*er* reads with no SNP information are marked in yellow. Col, *Arabidopsis thaliana* Columbia ecotype; L*er*, *Arabidopsis thaliana* Landsberg *erecta* ecotype; SV, structural variant; TE, transposable element; SNP, single nucleotide polymorphism.

When mapping the resequencing reads from L*er* on the Col reference genome, some reads from TEs (Figure 1A, red arrow) can be misaligned to non-allelic positions (Figure 1A, blue arrow), leading to wrong variant calls [18]. We totally found 4588 artificial polymorphisms (4204 SNPs and 384 indels) due to transposition reads. For example, a deletion of TE (AT1TE12135, Chr1:3,728,969–3,730,413 bp) on L*er* was detected by inGAP-family when the distances between the paired reads flanking this region were significantly longer than the average length of DNA fragments in sequencing library. As its genome does contain this TE in an alternative position (Figure 1A), non-allelic reads are translocated into this region, resulting in more than 10 artificial polymorphisms (Figure 1B).

**Figure 2:**
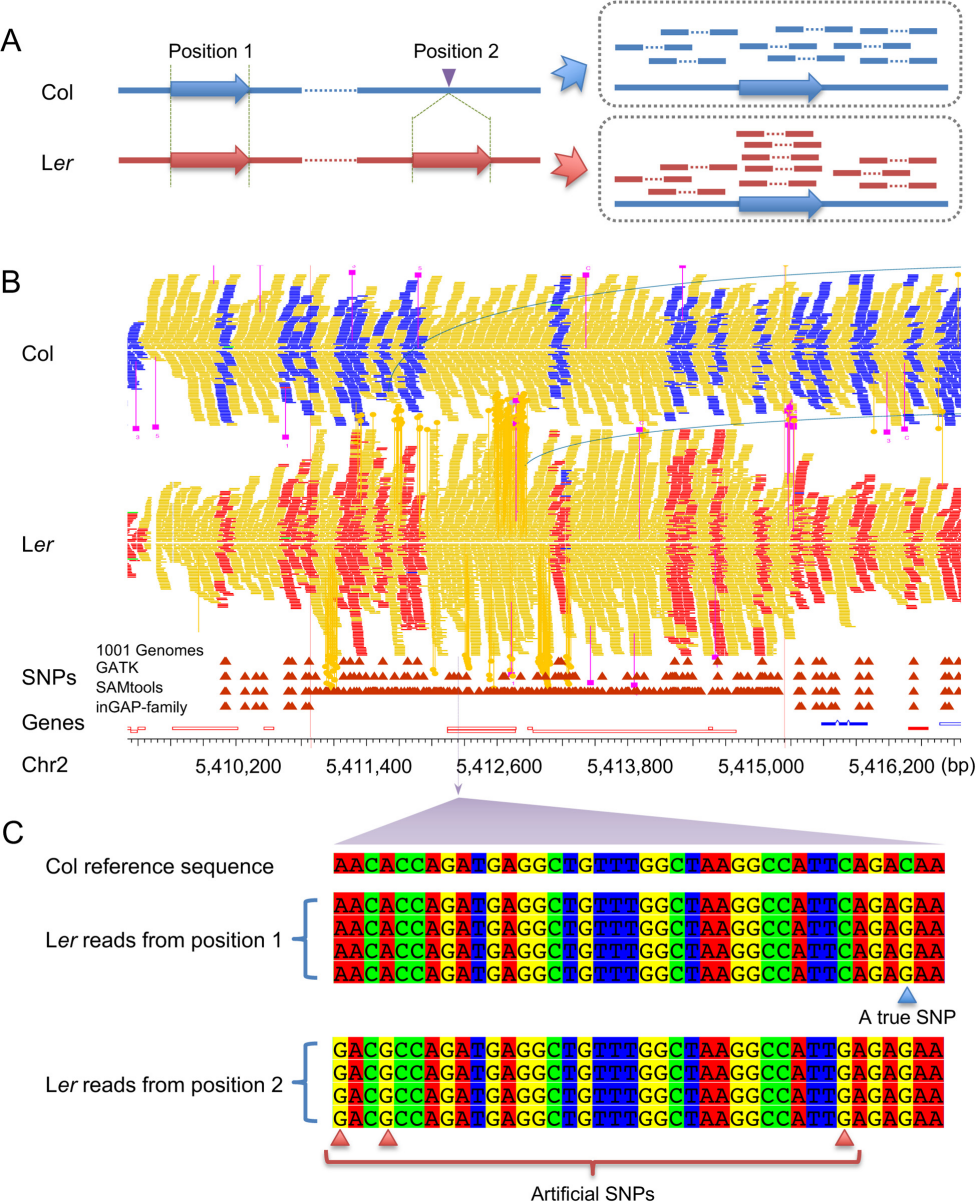
**Illustration of CNVs and their effects on allele ratio estimation** **A.** Display of the mapping pattern of paired-end reads on a CNV between Col and L*er*. **B.** An example of CNV between Col and L*er* (5,412,450–5,413,079 bp on Chr2 of Col) analyzed from resequencing data. **C.** L*er* reads in correct allelic position give one true SNP (blue triangle) while all the other three SNPs (red triangles) are artificial ones that result from reads from non-allelic positions. CNV, copy number variant.

CNVs can also lead to false positive variant calls or genotypes due to wrong mapping of non-allelic reads. We totally identified 1463 regions with singe copy in Col but two or more duplicates in L*er*, covering 2.7 Mb of the reference genome and bringing the artifacts of 6808 SNPs and 819 indels. For example, a TE AT2TE21915 (Chr2:5,412,450–5,413,079 bp) exhibiting single copy in Col genome (Figure 2A, blue arrow), has two copies in L*er* genome (Figure 2A, red arrows). Detailed alignments of L*er* reads (Figure 2B and C) illustrate that there is a slight divergence between the two paralogous sequences in L*er*: the true L*er* allele has only one substitution (from ‘C’ to ‘G’), while three artificial SNPs were called when non-allelic reads from the non-allelic duplicate were mapped to the same site. As there are 3903 annotated TE genes and more repetitive sequences according to the annotation of Col genome, obtaining reliable allelic polymorphisms is challengeable for genetic studies.

Another concern lies in small indels detected in tandemly repeated regions, within which different copy numbers of mono-, di-, tri- or longer nucleotides are commonly found between ecotypes. Many indels in tandem repeats are unable to be accurately detected by short reads, and unable to span the entire repetitive regions [18]. At nucleotides 2,842,132–2,842,152 on Chr1 of Col genome, there are 10 consecutive ‘AG’ dinucleotides, but only nine ‘AG’ dinucleotides (with a deletion of one ‘AG’) in L*er* genome (Figure S2). Two types of reads in this region are observed: some reads span the region and reveal the indel, while the other type of reads terminates within the tandem repeats without gap opening. The alignment of the second type of reads would subsequently be interpreted as evidence for an artificial second allele of a “heterozygosity” indel. As the Col genome contains 948,905 tandem repeats occupying 8% of total sequences, SNPs and small indels that are located in these regions could be falsely genotyped as “heterozygous” if reads that terminate in the region are taken into account.

### Obtaining high-confidence molecular markers by filtering artificial SNPs from SVs and tandem repeats (Dataset 1)

We compared the performance of inGAP-family, SAMtools [12], and GATK [13] for polymorphism detection between *A. thaliana* ecotypes Col and L*er*. A real resequencing data set of L*er* genome [20], including 76 million 2 × 100 paired-end reads (Dataset 1, see Method), was adopted to be compared with Col reference genome. A list of polymorphisms between Col and L*er*, including 461,070 SNPs and 120,254 small indels (from 1 bp to 10 bp), provided by 1001 Genomes Consortium [31], [32] through whole genome comparison, was adopted for evaluating variations predicted by these methods.

After removing those false predictions due to misalignment of non-allelic reads in SV regions and other factors (Table S1), inGAP-family identified 578,479 SNPs from 636,470 candidates based on Bayesian model [24]. First, a total of 7880 large-scale SVs, including 5777 deletions, 1463 duplications, 136 transpositions, 13 inversions, and 491 complex SVs were detected by inGAP-sv [25]. Among them, deletion regions produced 11,066 artificial SNP calls, because many deleted regions in L*er* have similar sequences in different loci causing false mapping of non-allelic short reads on reference genome. Furthermore, 4204, 6808, and 6823 artificial SNPs associated with transpositions, duplications, and complex SVs, respectively, were then removed from the final SNP list. Second, the read depth and allele ratio for each polymorphism were examined for recognizing artificial or unreliable SNP calls. In addition, 13,746 SNPs were ignored due to insufficient number of supported reads or abnormal coverage of depths. Similarly, 100,926 of 115,495 small indel candidates were retained by filtering out those located in various types of SVs and tandemly repeated regions.

**Figure 3:**
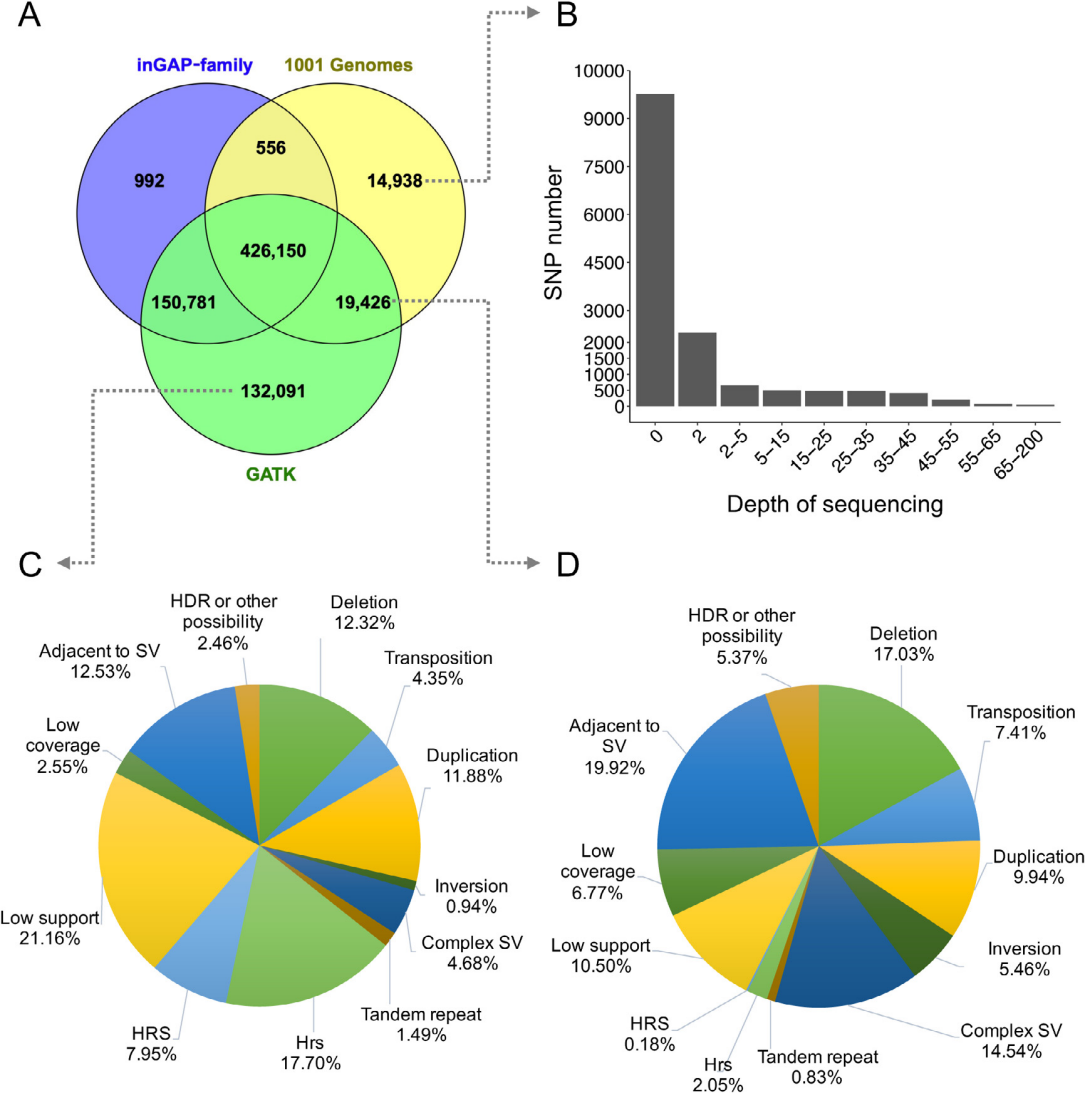
**Comparison of SNPs predicted by inGAP-family, GATK, and 1001 Genomes** **A.** A Venn diagram showing the numbers of SNPs uncovered by the three algorithms. **B.** Distribution of read depth of resequencing data of L*er* on SNPs missed by both inGAP-family and GATK. The 14,938 SNPs provided by 1001 Genomes are based on whole-genome comparison, while most of them have no sequence coverage on data adopted by inGAP-family and GATK, suggesting possibility of rescuing these SNPs when more data are available. **C.** Reinterpretation of 132,091 SNP calls by GATK with various factors. “hrs” refers to regions containing 20% or more reads with mapping quality lower than 20, “HRS” refers to regions with 50% or more non-uniquely mapped reads, and “HDR” denotes highly divergent regions (50% or more mutations in a 20-bp window). **D.** Classification of 19,426 SNPs uncovered by both GATK and 1001 Genomes with various factors. Most of these SNPs are within or adjacent to different types of SVs.

A list of 461,070 SNPs is given by 1001 Genomes Consortium between L*er* and Col ecotypes [31], [32], slightly lower than the 578,479 SNPs obtained by inGAP-family, while GATK gives a high number of 728,448 SNPs (Figure 3A). Notably, 426,150 of the 461,070 SNPs of 1001 Genomes Consortium (92.4%) were also reported by both inGAP-family and GATK, while 14,938 SNPs (3.2%) were missed by both methods, as the majority of them (9766 SNPs, 65.4%, Figure 3B) were not covered by any reads of the resequencing data of L*er* (SRX202247). Furthermore, compared with 992 algorithm-specific SNPs reported by inGAP-family, GATK reported 132,091 additional SNPs supported by neither 1001 Genomes Consortium nor inGAP-family (Figure 3A). Further examination on genome context of these 132,091 SNPs revealed that they were associated with large-scale SVs including deletions (12.32%), transpositions (4.35%), duplications (11.88%), and other complex SVs (4.68%), or in up-/down-stream regions flanking SVs (12.53%), or in heterochromatin regions with highly repeated sequences (25.65%, HRS and Hrs), or lack of sufficient sequence support (21.16%, see Method) or coverage (2.55%), or other possibilities (Figure 3C). Moreover, a careful examination on the 19,426 SNPs reported by both 1001 Genomes Consortium and GATK but not by inGAP-family (Figure 3A) revealed that the majority of them (74.30%) were located in or adjacent to large-scale SVs (54.38% and 19.92%, respectively), 17.27% of them lacked sufficient support or coverage (10.50% and 6.77%, respectively), and 2.23% of them lied within highly repeated regions (Figure 3D). Therefore, it is of essential importance to consider the effects of SVs and examine the repeated regions for accurate prediction of polymorphisms. In addition, as the L*er* genome is not fully assembled and covers 96% of the Col genome [32], many SNPs detected from the resequencing data by the two methods are missed in genome comparison (Figure 3A). Compared with inGAP-family and GATK [13], SAMtools [12] reported more variant callings (773,939 SNPs), which contained many artificial variants detected with similar effects by wrong mapping of non-allelic sequences (Figure S3; Table S1).

Besides the effects of large-scale SVs on variant detection, artificial indels due to incorrect gap opening of read mapping on tandemly repeated regions also account for a large number of false polymorphisms and must be removed before being further applied to genetic studies (Figure S2; Table S1). We identified a list of 948,929 tandem repeats with the unit length ranging from 1 bp to 24 bp on the Col reference genome using Tandem Repeats Finder [26], and employed them as input for SNP detection of inGAP-family. Thousands of false positive polymorphism callings contributed by short reads not spanning entire tandemly repeated regions are avoided (Table S1).

In summary, inGAP-family provides highly confident molecular markers between Col and L*er* ecotypes than previous studies [20], [29], with an average distance of 206 bp between adjacent SNPs. Application of inGAP-family on more species can provide better resolution on studies’ sensitive to the quality of polymorphisms.

### Detection of recombination events by inGAP-family (Dataset 1)

Meiotic recombination is an essential process that inflicts double strand breaks (DSBs) on the genome, and plays a critical role in ensuring the proper segregation of chromosomes [30], [33]. DSBs are repaired by either CO or GC events [18], [30]. Although genome-scale studies of COs and GCs have been performed in many species, *e.g.*, human [33], rice [34], yeast [35], [36], *Arabidopsis* [18], [20], [29], [30], and honey bee [37], few tools are developed to detect GCs directly from high-throughput sequencing data, as it is challengeable to detect GCs associated with allelic changes at specific polymorphic sites instead of large-scale genotype exchanges on COs. Consequently, artificial SNPs due to mapping of non-allelic sequences related to SVs and other genome complexities would lead to false prediction of GCs with two orders of magnitude or more than expected [18].

**Figure 4:**
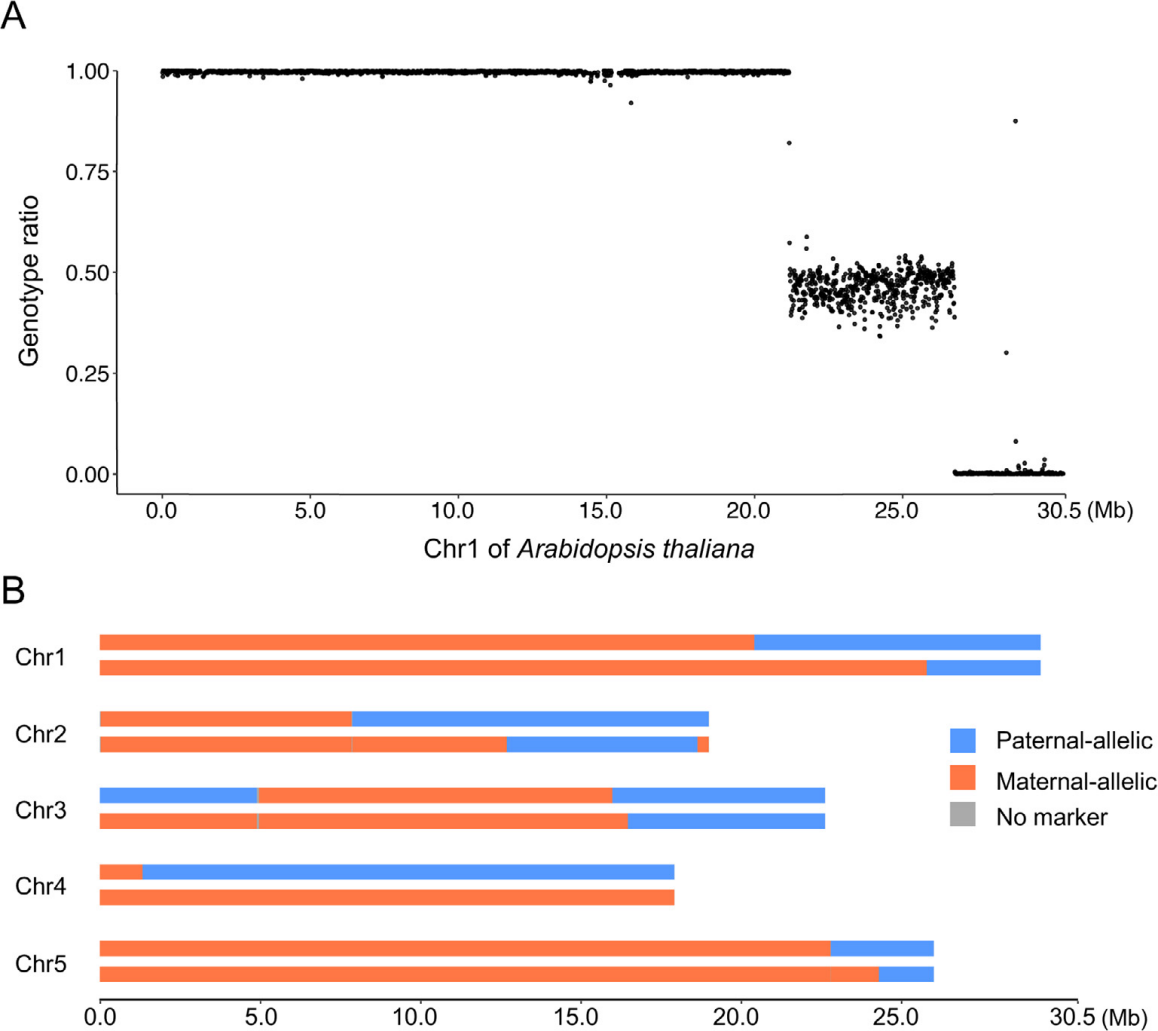
**Genotyping of a meiotic progeny of Col and L*er* by using SNPs predicted by inGAP-family on resequencing data of L*er*** **A.** Display of allelic ratios of SNP windows on Chr1. It was genotyped as L*er* homozygous (711–21,171,108 bp), heterozygous (21,171,909–26,746,175 bp), and Col homozygous (26,746,990–30,424,791 bp), indicating two CO events in Chr1. **B.** Genotype details of all five chromosomes of the progeny genome. For each chromosome, two rows denote genotypes of two chromatids, respectively. CO, crossover.

inGAP-family distinguishes non-allelic variants from allelic polymorphisms by examining effects of large indels, CNVs, and other types of SVs, and thus provides an accurate and convenient way to detect COs and GCs directly from resequencing short reads of meiotic progeny genomes. Here, we adopted one F_2_ plant hybridized from Col and L*er* to evaluate the performance of inGAP-family. Short reads of the F_2_ plant were aligned against the Col reference genome using BWA [28], and those reads with mapping quality score higher than 20 were used for genotyping when covering any of the 578,479 filtered SNPs. Potential COs should be identified prospectively prior to GC detection as COs are associated with exchange of flanking regions. The whole genome of the F_2_ plant was split into 11,637 sliding windows (size of 20 kb with step of 10 kb), and allele ratio was estimated on each window for genotyping (see Method). As a result, for example, Chr1, containing 2972 windows, was divided into three continuous blocks representing different genotypes: the first 20.2 Mb of Chr1 was genotyped as L*er* homozygous region, the next 5.3 Mb region was heterozygous, and the last 3.5 Mb region was Col homozygous (Figure 4A). Allele ratio at each SNP locus adjacent to the two identified CO events was carefully examined, to ascertain the exact borders of the COs for Chr1 (21,171,108–21,171,909 bp and 26,746,175–26,746,990 bp). Similarly, a total of nine COs were identified in the other four chromosomes (Figure 4B, Figure S4; Table S2).

Furthermore, when a SNP has a different genotype from those of the neighboring SNPs, it would be labeled as a converted SNP for a potential candidate of a GC event. Continuous converted SNPs with a distance shorter than 1 kb apart from each other were grouped into one GC event. In conclusion, we totally identified five GC candidates in the genome of the progeny plant (Table S3), consistent with a prior study [18] that most of DSBs during meiosis are repaired using sister chromosomes instead of homolog chromosomes as templates and few of them are able to be recognized as GC events.

### Genetic mapping and identification of causal mutations (Dataset 2)

**Figure 5:**
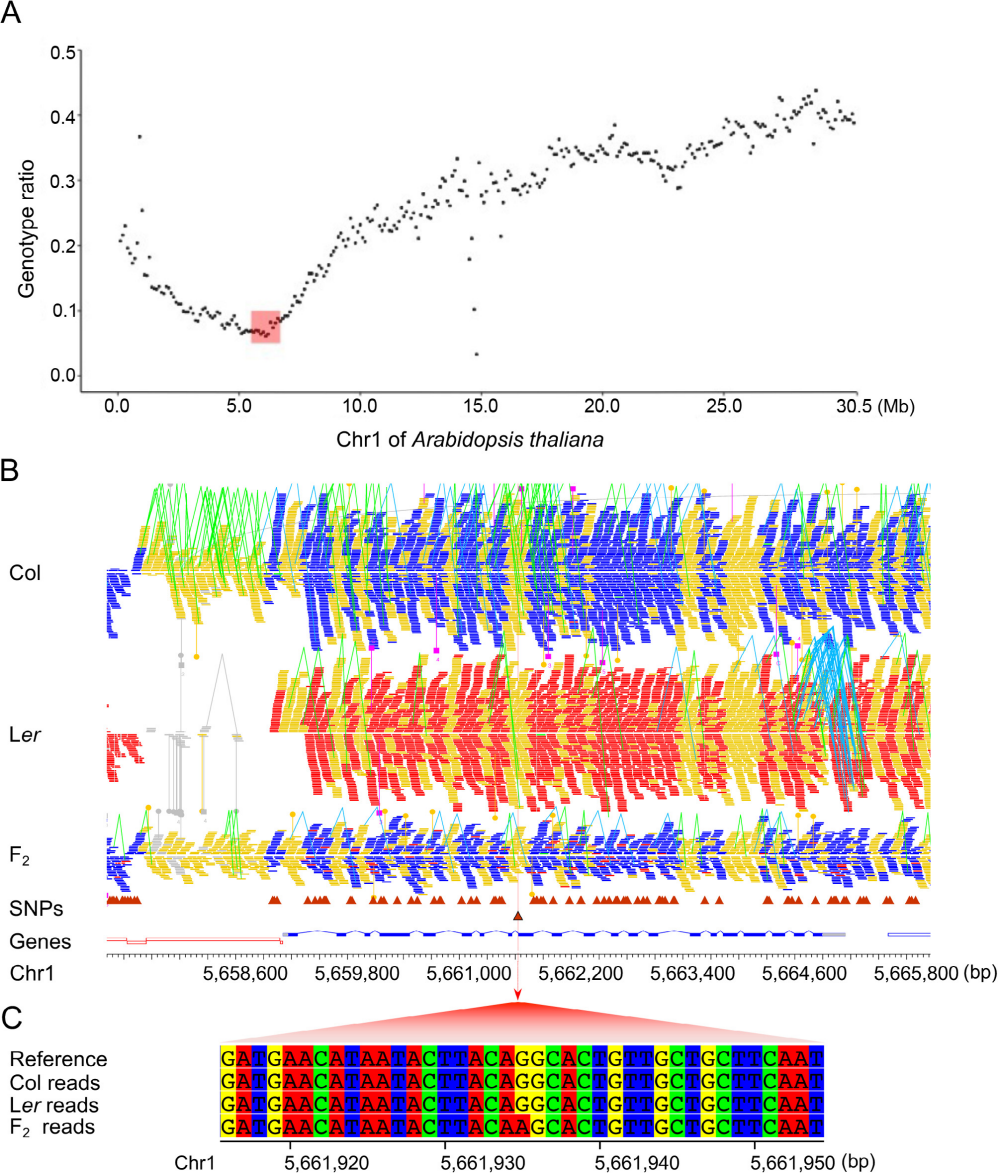
**Identification of causal mutations from pooled genome sequencing of 100 F_2_ progenies hybridized from the wild-type Col and a L*er* EMS mutant** **A.** The distribution of allelic ratios of SNP windows on Chr1. Allele ratios are estimated on sliding windows of 200 kb with step of 100 kb. The region including candidate mutations is marked by a pink box. **B.** Mapping details of short reads from Col, L*er*, and F_2_ plants flanking the position of 5,661,935 bp on Chr1. Reads genotyped as Col and L*er* are colored in blue and red, respectively. **C.** Display of the causal mutation (from ‘G’ to ‘A’) associated with salt-tolerant phenotype. EMS, ethane methyl sulfonate.

High-throughput sequencing technology has been widely applied for detection of QTLs, as well as causal mutations induced by EMS, T-DNA insertion [9], [14], [15], [16], *etc.*, providing an efficient and effective way to identify these interested mutations. When high-confidence genomic markers are obtained after filtering out false polymorphisms, and each of the sliding windows (size of 200 kb with step of 100 kb, as mentioned above) is genotyped, inGAP-family can easily identify novel mutations, including substitutions, small indels, and large-scale SVs, within windows with expected allelic ratios. To test the performance of inGAP-family upon real case studies, we sequenced a DNA pool of 100 F_2_ progenies with salt-tolerance phenotype hybridized from an EMS-induced Col mutant and a wild-type L*er* ecotype (see Method). A total of 44 million paired-end reads (2 × 100 bp) were obtained on an Illumina platform, providing a sequencing depth of 25× on average. The reference genome was partitioned into 1167 windows, for which genotype was determined according to the allelic ratio. A region in Chr1 (5,529,601–6,758,400 bp) was found to exhibit the expected allele ratio (Figure 5A, Figure S5), and was considered as a candidate region carrying the causal mutation.

inGAP-family detected eight novel mutations within the candidate region in F_2_ progenies comparing with parental genomes (Table S4). Among them, one mutation was located in splice site of *ABA3* (AT1G16540), and thus affected the alternative splicing of its transcript (alternative acceptors on the 9th exon), whose product functions in the abscisic acid biosynthesis pathway (Figure 3B and C, Figure S6). One synonymous and four non-synonymous substitutions were found on five genes, respectively, while the other two mutations were located in introns (Table S4). The causal mutation on *ABA3* was confirmed by complementation of *ABA3* into mutant plants where the phenotype was rescued. We also applied SHOREmap [14] with parameters as described by Sun et al. [38] upon the same data for comparison. The region with causal mutations estimated by SHOREmap was of ∼ 1 Mb (5,279,999–6,279,999 bp) on Chr1 (Figure S7), within which a total of 109 SNPs associated with 44 genes were reported as candidates. These results demonstrate that inGAP-family could advantageously provide a single-base resolution in the identification of a causal point mutation induced by EMS or similar methods.

## Discussion

An essential concern of the procedure of inGAP-family is to remove artificial variants brought by non-allelic sequences from large-scale SVs and other types of repetitive sequences. SVs including large deletions, insertions, and duplications are detected by inGAP-sv, which exhibits better performance than other popularly used methods (Table S5) and is already integrated into the pipeline of inGAP-family. Short tandemly repetitive sequence is predicted by Tandem Repeat Finder [26] and is taken as input of inGAP-family for filtering out artificial SNPs or small indels. However, the complex nature of eukaryotic genomes leads to many SVs remaining uncovered, especially among heterochromatin regions including centromeres and telomeres, thus keeping many artificial polymorphisms undiscovered in related analyses. In addition, organisms with recent polyploidization, commonly found in many crops, *e.g.*, wheat (*Triticum aestivum*), peanut (*Arachis hypogaea*), potato (*Solanum tuberosum*), rapeseed (*Brassica napus*), and sugarcane (*Saccharum spontaneum* L.), contain two or more sets of chromosomes with high sequence similarity, bringing great challenges for short read mapping and subsequent SV identification. These SVs are difficult to be accurately identified by short NGS reads. This problem can be partially overcome by incorporating with data of the third-generation high-throughput sequencing platforms, *e.g.*, Pacific Biosciences [39] and Oxford Nanopore Technologies [40], which generate sequences with length of 10,000 bp or longer and thus greatly facilitate the detection of complex SVs [41], [42]. The integration of more types of high-throughput sequencing data can provide more accurate polymorphisms as molecular markers.

*De novo* mutations (DNMs) make important contributions to genome diversity across many species [43]. These mutations are largely connected with human genetic diseases, as they are highly disruptive to gene function and affect domains of developmental genes [44]. Trio-based whole-genome sequencing platform greatly facilitates the detection of DNMs in rare genetic diseases [44], [45], [46], and many bioinformatic methods have been developed for identifying DNMs from NGS data, *e.g.*, PolyMutt [47], DeNovoGear [48], DNMFilter [49], Triodenovo [50], and mirTrios [51]. By comparing with reference genomes, DNMs are called for each individual, and then evaluated in a parent–offspring context [46], [48], [49]. Naturally, accurate detection of DNMs depends on filtering of artificial calls from false alignment of non-allelic sequences from SVs [18], [49]. Automatic and accurate detection of DNMs from trio- or family-based resequencing data could be integrated to inGAP-family and calls for further study.

## Conclusion

In summary, the advances of NGS technologies and computational methods for variation discovery and genotyping have greatly facilitated the identification of DNA polymorphisms and large-scale genomic variants, and promoted many genetic studies depending on molecular markers. Although many tools are available for variant calling [12], [13], [17], [24], [25], few of them consider filtering of artificial variants due to false alignment of non-allelic sequences from SVs or other types of genome complexities, which brings difficulties to many genetic studies, *e.g.*, analysis of meiotic recombination, genetic mapping, and detection of causal mutations. In this study, we employ an integrated comprehensive strategy, inGAP-family, to predict and refine SNPs and small indels directly from NGS sequence mappings or from polymorphisms of third-party software (with VCF format), *e.g.*, GATK [13] and SAMtools [12]. These variants are then adopted by inGAP-family as high-quality molecular markers, providing better resolution to identify meiotic recombination loci or mutations responsible for the phenotypic divergences than directly utilizing polymorphism callings from other methods. inGAP-family requires more computational time and memory than SAMtools [12] but less than GATK [13], and it is affordable on many servers for identifying and filtering SNPs (Table S6). The graphic interface of inGAP-family allows users to visualize mapping and alignment details of short reads associated with specific genome polymorphisms, and examine meiotic recombination events or causal mutations for further experimental verification.

## Code availability

inGAP-family is written in platform-independent Java programming language, and can be freely accessed at https://sourceforge.net/projects/ingap-family/.

## Data availability

Sequencing data in this study have been deposited in the Genome Sequence Archive [52] at the National Genomics Data Center, Beijing Institute of Genomics, Chinese Academy of Sciences / China National Center for Bioinformation (GSA: CRA002018), and are publicly accessible at https://ngdc.cncb.ac.cn/gsa.

## CRediT author statement

**Qichao Lian:** Methodology, Software, Formal analysis, Writing - original draft, Visualization. **Yamao Chen:** Investigation, Writing - review & editing. **Fang Chang:** Validation. **Ying Fu:** Validation. **Ji Qi:** Supervision, Conceptualization, Software, Writing - review & editing. All authors have read and approved the final manuscript.

## Competing interests

The authors have declared no competing interests.

## Supplementary material

Supplementary Figure S1 **The pipeline of variant calling, filtering and multiple applications by inGAP-family**

Supplementary Figure S2 **Illustration of artificial indel callings due false gap opening in tandemly repeated regions**

Supplementary Figure S3 **Comparison of SNPs predicted by inGAP-family, GATK, Samtools and 1001 Genomes**

Supplementary Figure S4 **Genotyping of a meiotic progeny of Col and L*er* by using SNPs predicted by inGAP-family on re-sequencing data of L*er***

Supplementary Figure S5 **Allele ratio estimation for pooled genome sequencing of 100 F2 progenies for identification of causal mutations by using inGAP-family with sliding windows of 200 kb and step of 100 kb**

Supplementary Figure S6 **The validation of an EMS induced mutation on *ABA3* gene (AT1G16540) by using qRT-PCR**

Supplementary Figure S7 **Allele frequencies of pooled genome sequencing of 100 F2 progenies estimated by SHOREmap**

Supplementary Table S1 **A summary of SNPs/indels by 1001 Genomes, GATK and Samtools and their distributions on various factors between two *Arabidopsis thaliana* ecotypes, Col and L*er***

Supplementary Table S2 **A list of predicted crossover borders on a F2 progeny hybridized from two *Arabidopsis thaliana* ecotypes, Col and L*er* (Dataset 1)**

Supplementary Table S3 **A list of predicted gene conversions on a F2 progeny hybridized from two *Arabidopsis thaliana* ecotypes, Col and L*er* (Dataset 1)**

Supplementary Table S4 **A list of mutations discovered in a pooled genome sequencing of 100 F2 progenies hybridized from a wild-type Col and a L*er* mutant induced by EMS (Dataset 2)**

Supplementary Table S5 **A comparison of large deletions by inGAP-family, BreakDancer and CNVnator on a whole-genome resequencing dataset of the ecotype L*er* of *Arabidopsis thaliana* (Dataset 1)**

Supplementary Table S6 **A comparison of computational consumption by inGAP-family, GATK and Samtools on two distinct whole-genome resequencing datasets of *Arabidopsis thaliana* and *Homo sapiens***
