# Supplementary material for: inGAP-family: Accurate Detection of Meiotic Recombination Loci and Causal Mutations by Filtering Out Artificial Variants due to Genome Complexities
Source: Genomics Proteomics Bioinformatics. 2021 Mar 10;20(3):524–35. doi: 10.1016/j.gpb.2019.11.014 (PMC9801030; doi:10.1016/j.gpb.2019.11.014)
Supplement: Supplementary Figure S1 — The pipeline of variant calling, filtering and multiple applications by inGAP-family [file mmc1.pdf]

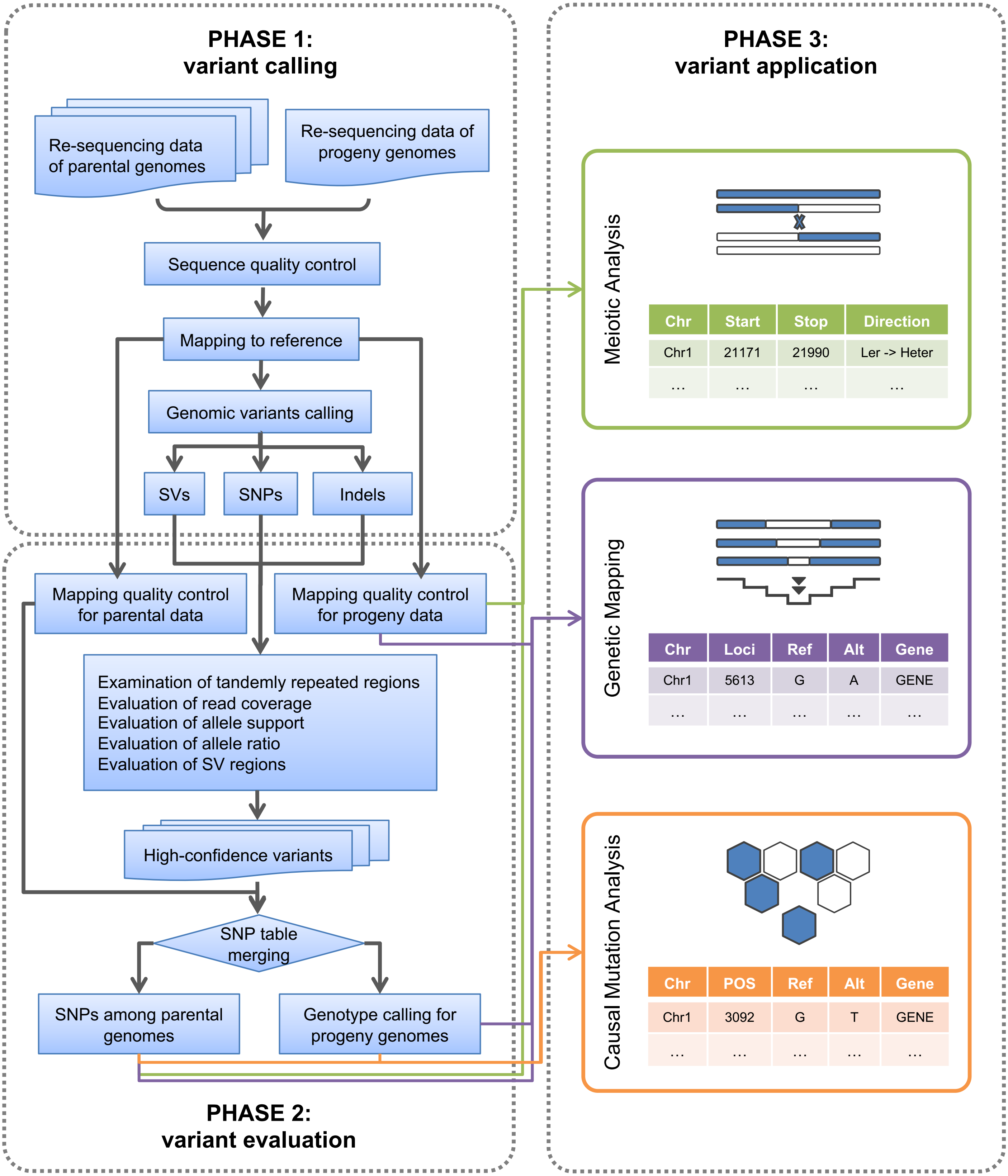
PHASE 1:
variant calling
Re-sequencing data of parental genomes
Re-sequencing data of progeny genomes
Sequence quality control
Mapping to reference
Genomic variants calling
SVs
SNPs
Indels
Mapping quality control for parental data
Mapping quality control for progeny data
Examination of tandemly repeated regions
Evaluation of read coverage
Evaluation of allele support
Evaluation of allele ratio
Evaluation of SV regions
High-confidence variants
SNP table merging
SNPs among parental genomes
Genotype calling for progeny genomes
PHASE 2:
variant evaluation
PHASE 3:
variant application
Meiotic Analysis
Chr | Start | Stop | Direction
Chr1 | 21171 | 21990 | Ler -> Heter
… | … | … | …
Genetic Mapping
Chr | Loci | Ref | Alt | Gene
Chr1 | 5613 | G | A | GENE
… | … | … | … | …
Causal Mutation Analysis
Chr | POS | Ref | Alt | Gene
Chr1 | 3092 | G | T | GENE
… | … | … | … | …
