# Supplementary material for: inGAP-family: Accurate Detection of Meiotic Recombination Loci and Causal Mutations by Filtering Out Artificial Variants due to Genome Complexities
Source: Genomics Proteomics Bioinformatics. 2021 Mar 10;20(3):524–35. doi: 10.1016/j.gpb.2019.11.014 (PMC9801030; doi:10.1016/j.gpb.2019.11.014)
Supplement: Supplementary Figure S2 — Illustration of artificial indel callings due false gap opening in tandemly repeated regions [file mmc2.pdf]

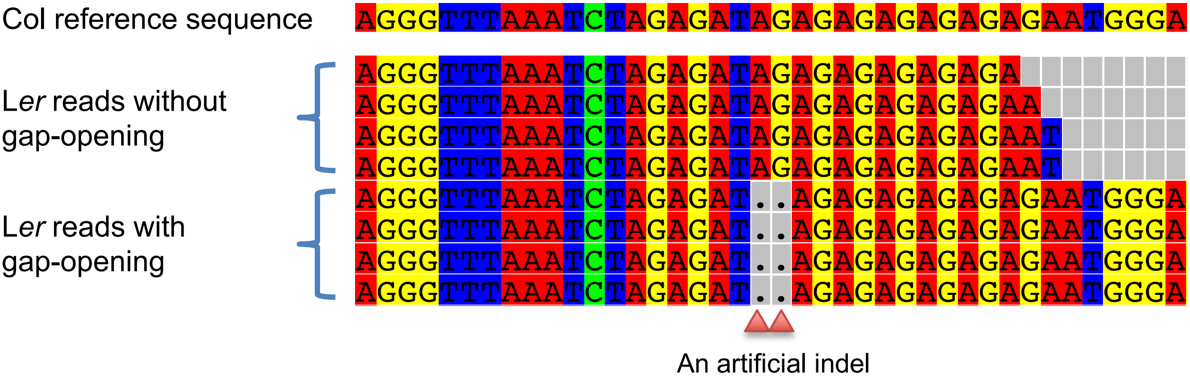

Col reference sequence
AGGGTTTAAATCTAGAGATAGAGAGAGAGAGAGAATGGGA
Ler reads without gap-opening
AGGGTTTAAATCTAGAGATAGAGAGAGAGAGA
AGGGTTTAAATCTAGAGATAGAGAGAGAGAGAA
AGGGTTTAAATCTAGAGATAGAGAGAGAGAGAAT
AGGGTTTAAATCTAGAGATAGAGAGAGAGAGAAT
Ler reads with gap-opening
AGGGTTTAAATCTAGAGAT..AGAGAGAGAGAGAATGGGA
AGGGTTTAAATCTAGAGAT..AGAGAGAGAGAGAATGGGA
AGGGTTTAAATCTAGAGAT..AGAGAGAGAGAGAATGGGA
AGGGTTTAAATCTAGAGAT..AGAGAGAGAGAGAATGGGA
An artificial indel
