# Supplementary figures and images for: inGAP-family: Accurate Detection of Meiotic Recombination Loci and Causal Mutations by Filtering Out Artificial Variants due to Genome Complexities
Source: Genomics Proteomics Bioinformatics. 2021 Mar 10;20(3):524–35. doi: 10.1016/j.gpb.2019.11.014 (PMC9801030; doi:10.1016/j.gpb.2019.11.014)

**1001 Genomes**

**GATK**

**inGAP-family**

**Samtools**

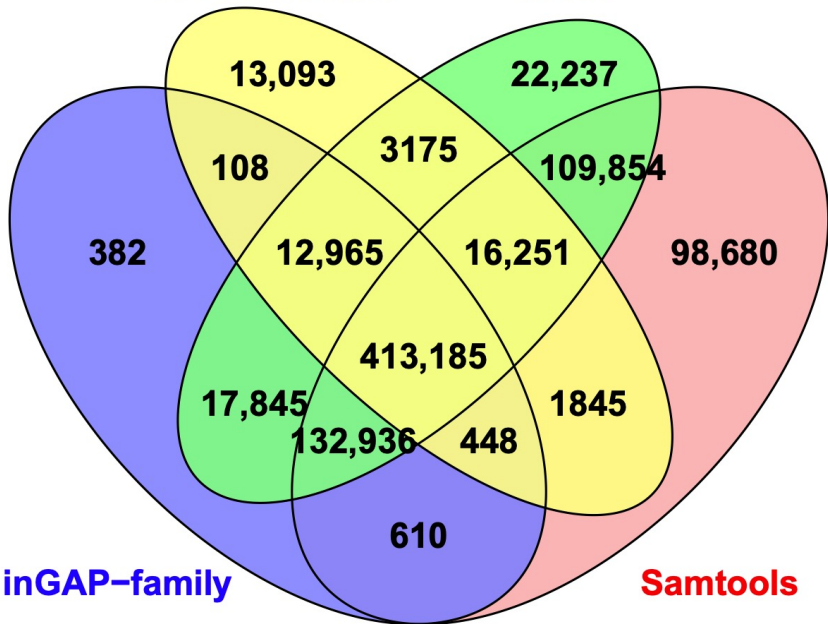

Supplement: Supplementary Figure S3 — Comparison of SNPs predicted by inGAP-family, GATK, Samtools and 1001 Genomes [file mmc3.pdf]

A Chromosome 2

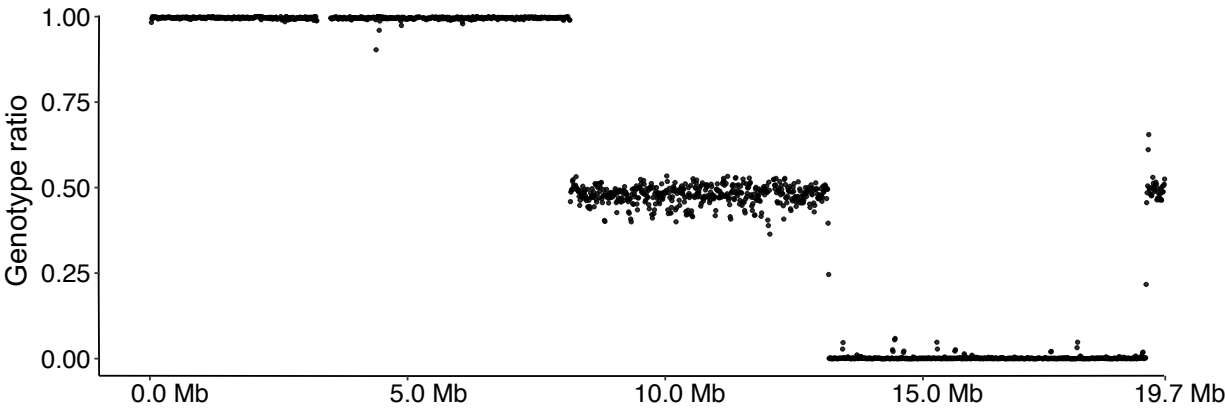

B Chromosome 3

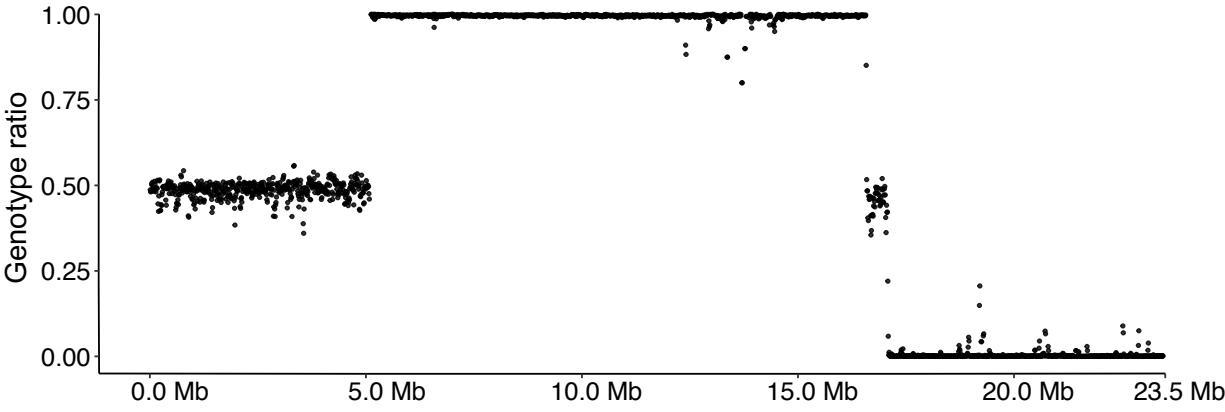

C Chromosome 4

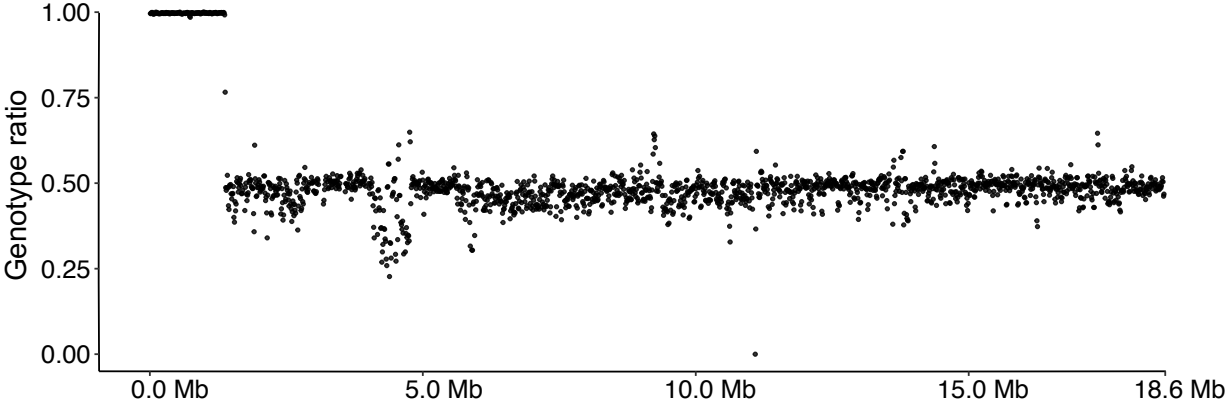

D Chromosome 5

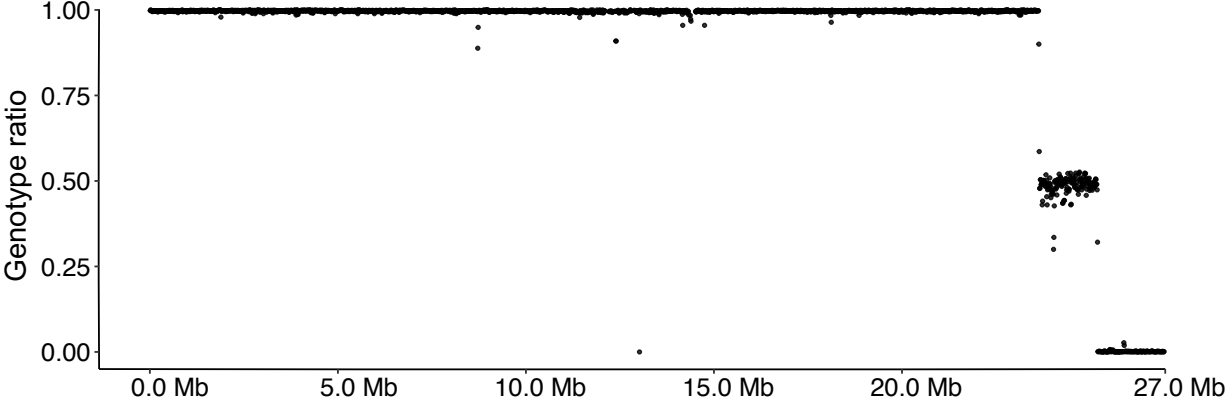

Supplement: Supplementary Figure S4 — Genotyping of a meiotic progeny of Col and Ler by using SNPs predicted by inGAP-family on re-sequencing data of Ler [file mmc4.pdf]

A Chromosome 2

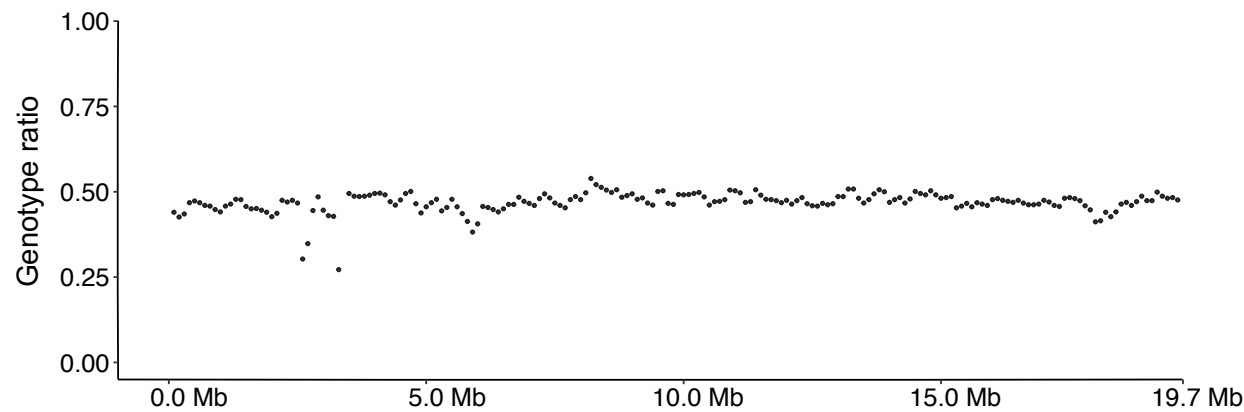

B Chromosome 3

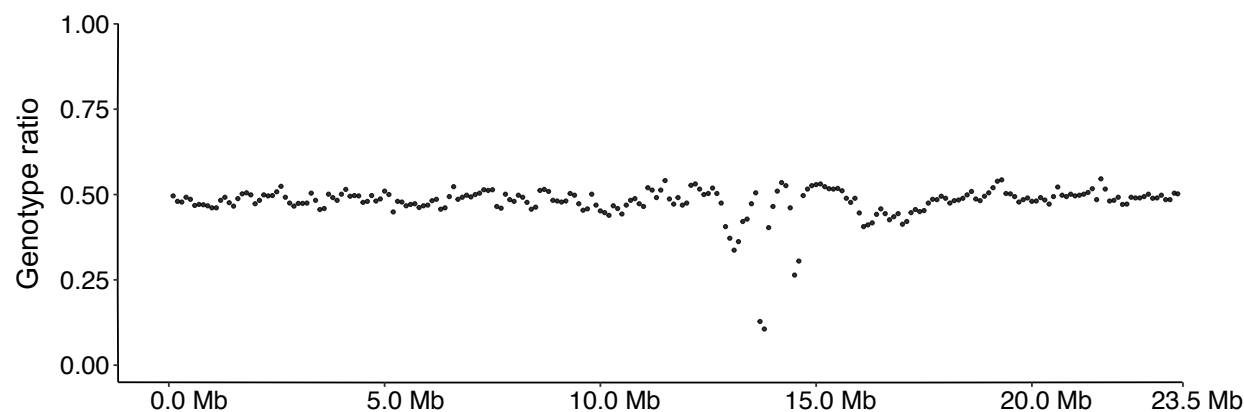

C Chromosome 4

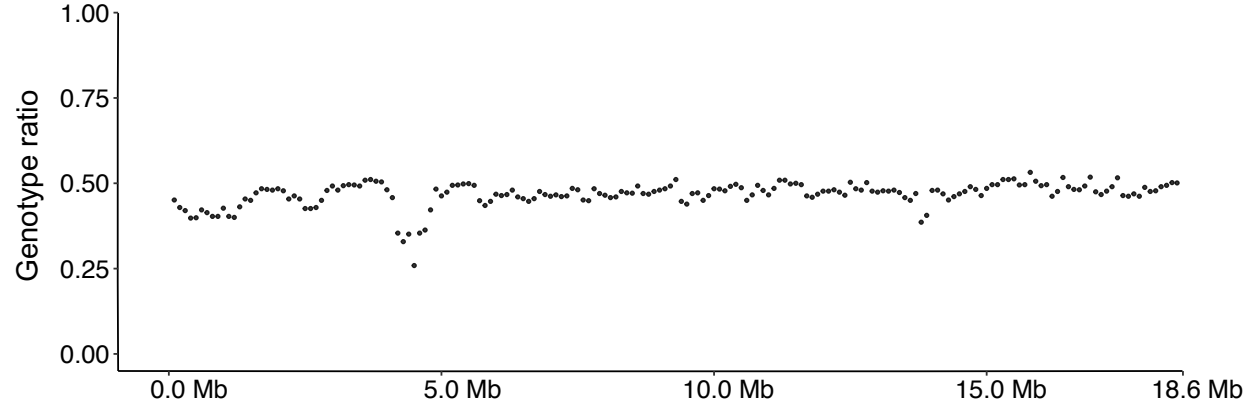

D Chromosome 5

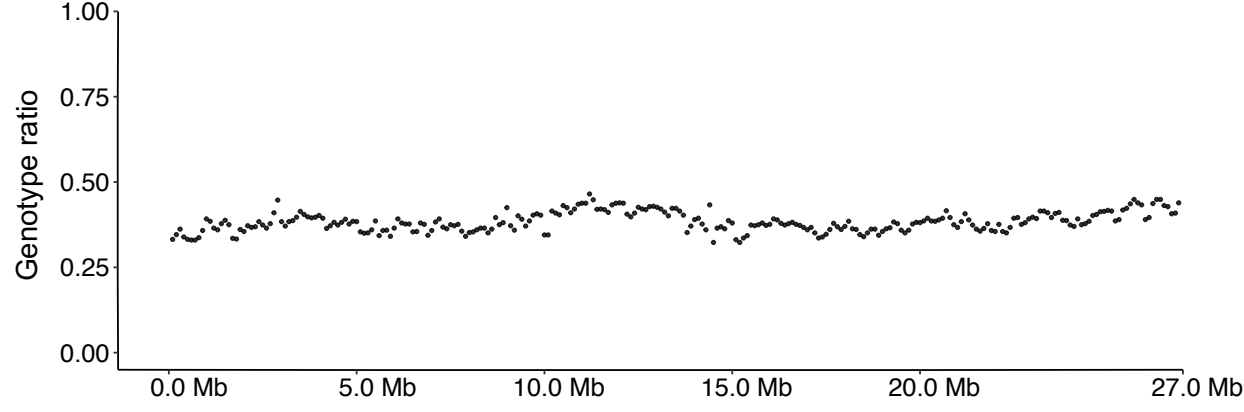

Supplement: Supplementary Figure S5 — Allele ratio estimation for pooled genome sequencing of 100 F2 progenies for identification of causal mutations by using inGAP-family with sliding windows of 200 kb and step of 100 kb [file mmc5.pdf]

## A Chromosome 1

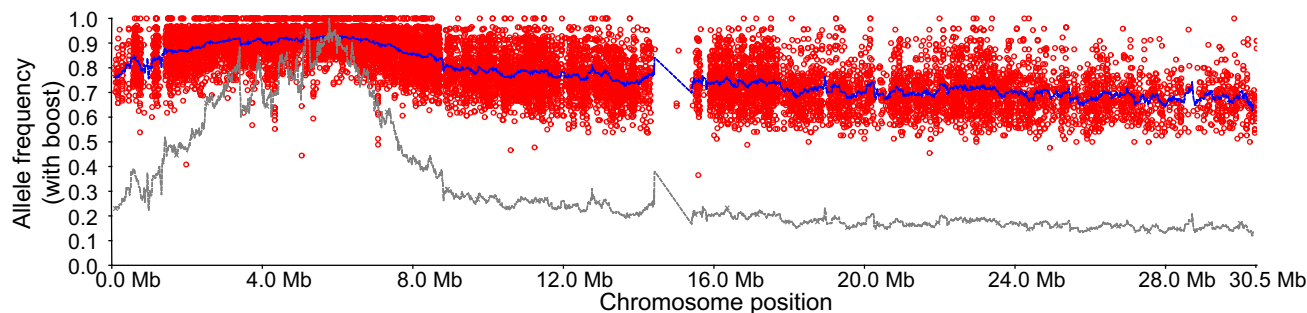

## B Chromosome 2

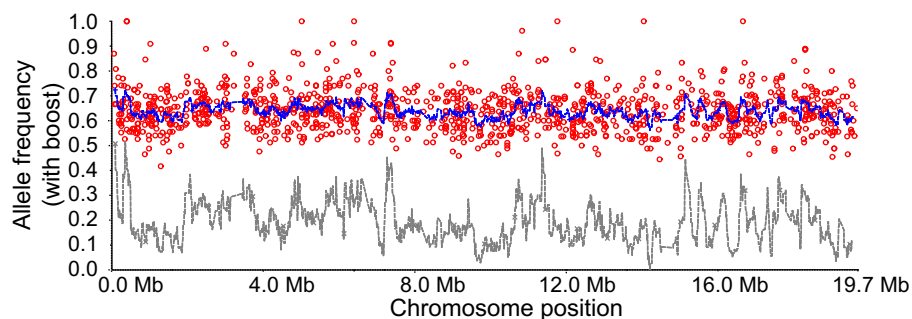

## C Chromosome 3

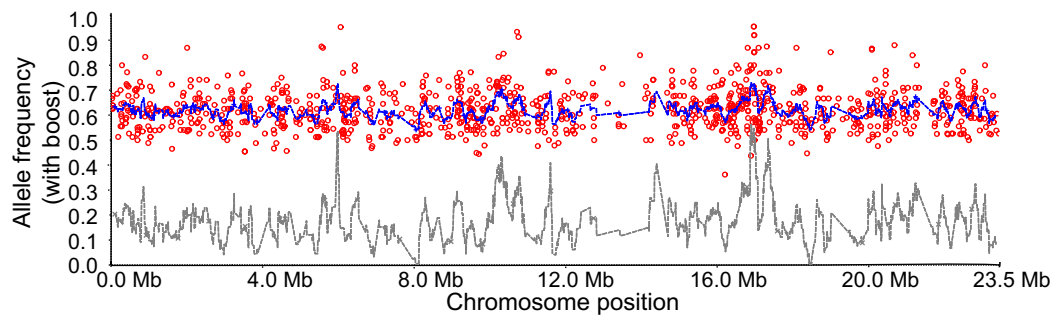

## D Chromosome 4

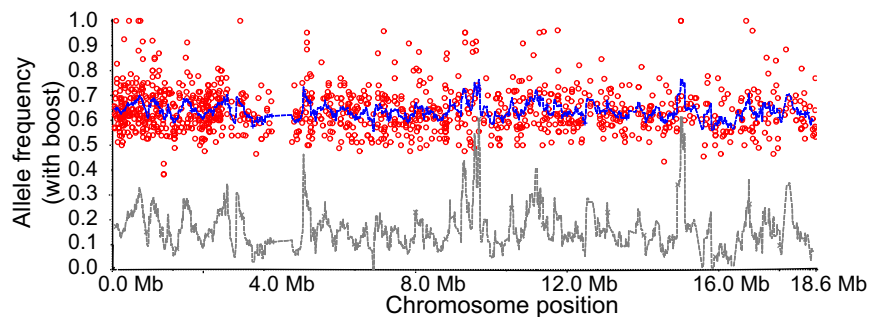

## E Chromosome 5

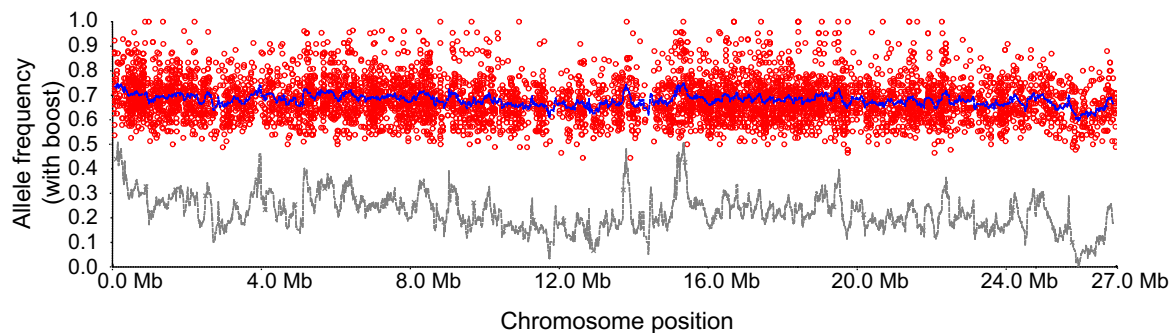

Supplement: Supplementary Figure S7 — Allele frequencies of pooled genome sequencing of 100 F2 progenies estimated by SHOREmap [file mmc7.pdf]
