# Supplementary material for: inGAP-family: Accurate Detection of Meiotic Recombination Loci and Causal Mutations by Filtering Out Artificial Variants due to Genome Complexities
Source: Genomics Proteomics Bioinformatics. 2021 Mar 10;20(3):524–35. doi: 10.1016/j.gpb.2019.11.014 (PMC9801030; doi:10.1016/j.gpb.2019.11.014)
Supplement: Supplementary Figure S6 — The validation of an EMS induced mutation on ABA3 gene (AT1G16540) by using qRT-PCR [file mmc6.pdf]

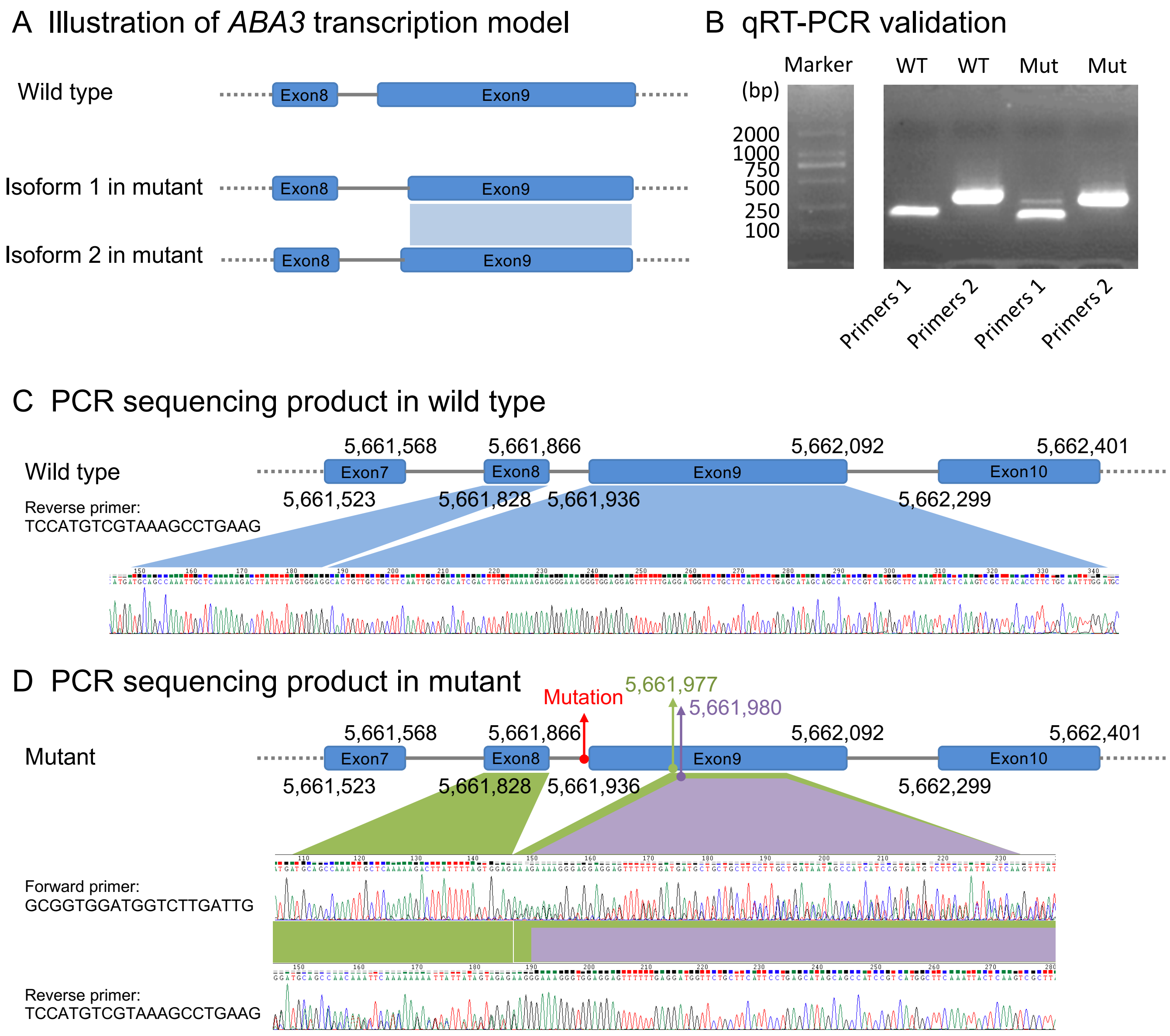
A Illustration of ABA3 transcription model
Wild type
Isoform 1 in mutant
Isoform 2 in mutant
Exon8
Exon9
B qRT-PCR validation
Marker
WT
WT
Mut
Mut
(bp)
2000
1000
750
500
250
100
Primers 1
Primers 2
Primers 1
Primers 2
C PCR sequencing product in wild type
Wild type
5,661,568
5,661,866
5,662,092
5,662,401
Exon7
Exon8
Exon9
Exon10
5,661,523
5,661,828
5,661,936
5,662,299
Reverse primer:
TCCATGTCGTAAAGCCTGAAG
D PCR sequencing product in mutant
Mutation
5,661,977
5,661,980
Mutant
5,661,568
5,661,866
5,662,092
5,662,401
Exon7
Exon8
Exon9
Exon10
5,661,523
5,661,828
5,661,936
5,662,299
Forward primer:
GCGGTGGATGGTCTTGATTG
Reverse primer:
TCCATGTCGTAAAGCCTGAAG
