# Supplementary material for: inGAP-family: Accurate Detection of Meiotic Recombination Loci and Causal Mutations by Filtering Out Artificial Variants due to Genome Complexities
Source: Genomics Proteomics Bioinformatics. 2021 Mar 10;20(3):524–35. doi: 10.1016/j.gpb.2019.11.014 (PMC9801030; doi:10.1016/j.gpb.2019.11.014)
Supplement: Supplementary Table S1 — A summary of SNPs/indels by 1001 Genomes, GATK and Samtools and their distributions on various factors between two Arabidopsis thaliana ecotypes, Col and Ler [file mmc8.docx]

**Table S1**  **A summary of SNPs/indels by 1001 Genomes, GATK and Samtools and their distributions on various factors between two *Arabidopsis thaliana* ecotypes, Col and L*er***

| **Factors** | **SNPs** | | |  | **Indels** | | |
| --- | --- | --- | --- | --- | --- | --- | --- |
|  | **1001 Genomes** | **GATK** | **Samtools** |  | **1001 Genomes** | **GATK** | **Samtools** |
| Raw | 461070 | 728448 | 773939 |  | 120254 | 110892 | 94373 |
| Deletion | 4523 | 19585 | 24765 |  | 2498 | 1403 | 1214 |
| Transposition | 1521 | 7179 | 10529 |  | 174 | 455 | 501 |
| Duplication | 1961 | 17627 | 35532 |  | 495 | 925 | 1195 |
| Inversion | 1120 | 2300 | 2532 |  | 214 | 276 | 257 |
| Complex SV | 2999 | 9005 | 11142 |  | 3016 | 814 | 731 |
| Tandem repeat | 248 | 2132 | 4836 |  | NA | 903 | 439 |
| hrs ^1^ | 541 | 23775 | 33648 |  | 956 | 1119 | 1142 |
| HRS ^2^ | 132 | 10529 | 18124 |  | 454 | 369 | 411 |
| Low support | 9465 | 29995 | 21096 |  | NA | 8301 | 13287 |
| Low coverage | 3806 | 4682 | 1779 |  | NA | 460 | 1556 |
| PASS | 434754 | 601639 | 609956 |  | 112447 | 95867 | 73640 |
