# Supplementary material for: inGAP-family: Accurate Detection of Meiotic Recombination Loci and Causal Mutations by Filtering Out Artificial Variants due to Genome Complexities
Source: Genomics Proteomics Bioinformatics. 2021 Mar 10;20(3):524–35. doi: 10.1016/j.gpb.2019.11.014 (PMC9801030; doi:10.1016/j.gpb.2019.11.014)
Supplement: Supplementary Table S2 — A list of predicted crossover borders on a F2 progeny hybridized from two Arabidopsis thaliana ecotypes, Col and Ler (Dataset 1) [file mmc9.docx]

**Table S2 A List of predicted crossover borders on a F_2_ progeny hybridized from two *Arabidopsis thaliana* ecotypes, Col and L*er* (Dataset 1)**

| **Chr** | **Start** | **End** | **Direction** |
| --- | --- | --- | --- |
| Chr1 | 21171108 | 21171909 | Ler to Heterozygous |
| Chr1 | 26746175 | 26746990 | Heterozygous to Col |
| Chr2 | 8147481 | 8168512 | Ler to Heterozygous |
| Chr2 | 13163808 | 13164695 | Heterozygous to Col |
| Chr2 | 19330796 | 19330992 | Col to Heterozygous |
| Chr3 | 5079992 | 5152064 | Heterozygous to Ler |
| Chr3 | 16579762 | 16580826 | Ler to Heterozygous |
| Chr3 | 17082838 | 17083210 | Heterozygous to Col |
| Chr4 | 1379708 | 1379976 | Ler to Heterozygous |
| Chr5 | 23633305 | 23646480 | Ler to Heterozygous |
| Chr5 | 25197899 | 25199080 | Heterozygous to Col |
