# Supplementary material for: inGAP-family: Accurate Detection of Meiotic Recombination Loci and Causal Mutations by Filtering Out Artificial Variants due to Genome Complexities
Source: Genomics Proteomics Bioinformatics. 2021 Mar 10;20(3):524–35. doi: 10.1016/j.gpb.2019.11.014 (PMC9801030; doi:10.1016/j.gpb.2019.11.014)
Supplement: Supplementary Table S3 — A list of predicted gene conversions on a F2 progeny hybridized from two Arabidopsis thaliana ecotypes, Col and Ler (Dataset 1) [file mmc10.docx]

**Table S3 A list of predicted gene conversions on a F_2_ progeny hybridized from two *Arabidopsis thaliana* ecotypes, Col and L*er* (dataset 1)**

| **Chr** | **Site** | **Col** | **Ler** | **Type** | **Direction** |
| --- | --- | --- | --- | --- | --- |
| Chr3 | 3545989 | - | A | indel | Heterozygous to Col |
| Chr3 | 7133180 | T | C | SNP | Ler to Heterozygous |
| Chr4 | 8986595 | C | T | SNP | Heterozygous to Ler |
| Chr4 | 12358751 | T | C | SNP | Heterozygous to Col |
| Chr4 | 13651179 | A | T | SNP | Heterozygous to Ler |
