# Supplementary material for: inGAP-family: Accurate Detection of Meiotic Recombination Loci and Causal Mutations by Filtering Out Artificial Variants due to Genome Complexities
Source: Genomics Proteomics Bioinformatics. 2021 Mar 10;20(3):524–35. doi: 10.1016/j.gpb.2019.11.014 (PMC9801030; doi:10.1016/j.gpb.2019.11.014)
Supplement: Supplementary Table S4 — A list of mutations discovered in a pooled genome sequencing of 100 F2 progenies hybridized from a wild-type Col and a Ler mutant induced by EMS (Dataset 2) [file mmc11.docx]

**Table S4 A list of mutations discovered in a pooled genome sequencing of 100 F_2_ progenies hybridized from a wild-type Col and a L*er* mutant induced by EMS (dataset 2)**

| **Chr** | **Loci** | **Ref** | **Alt** | **Col** | **Ler** | **F2** | **Gene** | **Type** |
| --- | --- | --- | --- | --- | --- | --- | --- | --- |
| Chr1 | 5623167 | G | A | 48 | 67 | 21 | AT1G16470 | Intronic |
| Chr1 | 5661935 | G | A | 61 | 47 | 21 | AT1G16540 | Alternative splicing |
| Chr1 | 5689748 | G | A | 56 | 49 | 21 | AT1G16650 | Non-synonymous |
| Chr1 | 5703081 | G | A | 43 | 66 | 19 | AT1G16680 | Non-synonymous |
| Chr1 | 5852193 | G | A | 36 | 50 | 20 | AT1G17120 | Non-synonymous |
| Chr1 | 6103887 | G | A | 52 | 45 | 15 | AT1G17745 | Non-synonymous |
| Chr1 | 6104202 | G | A | 54 | 45 | 12 | AT1G17745 | Intronic |
| Chr1 | 6239778 | G | A | 63 | 52 | 20 | AT1G18140 | synonymous |
