# Supplementary material for: inGAP-family: Accurate Detection of Meiotic Recombination Loci and Causal Mutations by Filtering Out Artificial Variants due to Genome Complexities
Source: Genomics Proteomics Bioinformatics. 2021 Mar 10;20(3):524–35. doi: 10.1016/j.gpb.2019.11.014 (PMC9801030; doi:10.1016/j.gpb.2019.11.014)
Supplement: Supplementary Table S5 — A comparison of large deletions by inGAP-family, BreakDancer and CNVnator on a whole-genome resequencing dataset of the ecotype Ler of Arabidopsis thaliana (Dataset 1) [file mmc12.docx]

**Table S5**  **A comparison of large deletions by inGAP-family, BreakDancer and CNVnator on a whole-genome resequencing dataset of the ecotype L*er* of *Arabidopsis thaliana* (dataset 1)**

|  | **inGAP-family** | **BreakDancer** | **CNVnator** |
| --- | --- | --- | --- |
| Total predicted deletions (20 bp to 100 kb) | 4372 | 3657 | 1190 |
| Deletions shared by inGAP-family | N/A | 2702 (73.8%) | 1033 (86.8%) |
| Algorithm-specific deletions | 1362 | 984 | 361 |
| Marked as complex SVs by inGAP-family | N/A | 908 (92.3%) | 305 (84.5%) |
