# Supplementary material for: inGAP-family: Accurate Detection of Meiotic Recombination Loci and Causal Mutations by Filtering Out Artificial Variants due to Genome Complexities
Source: Genomics Proteomics Bioinformatics. 2021 Mar 10;20(3):524–35. doi: 10.1016/j.gpb.2019.11.014 (PMC9801030; doi:10.1016/j.gpb.2019.11.014)
Supplement: Supplementary Table S6 — A comparison of computational consumption by inGAP-family, GATK and Samtools on two distinct whole-genome resequencing datasets of Arabidopsis thaliana and Homo sapiens [file mmc13.docx]

**Table S6 A comparison of computational consumption by inGAP-family, GATK and Samtools on two distinct whole-genome resequencing datasets of *Arabidopsis thaliana* and *Homo sapiens***

|  | **inGAP-family** | **GATK** | **Samtools** |
| --- | --- | --- | --- |
| Sample | *Arabidopsis thaliana* (SRX202247, 8.4 Gb) | | |
| Time | 8h1m | 14h48m | 4h24m |
| Memory | 17.84 Gb | 20.28 Gb | 0.08 Gb |
|  |  |  |  |
| Sample | *Homo sapiens* (ERR251110, 10.8 Gb) | | |
| Time | 22h42m | 24h1m | 8h52m |
| Memory | 33.15 Gb | 23.35 Gb | 0.10 Gb |
